# Proteomic Profiling of Extracellular Vesicles in Inflammatory Bowel Diseases

**DOI:** 10.3390/ijms26020526

**Published:** 2025-01-09

**Authors:** Montse Baldán-Martín, Mikel Azkargorta, Ainhoa Lapitz, Lorena Ortega Moreno, Ibon Iloro, Samuel Fernández-Tomé, Ander Arbelaiz, Iraide Escobes, Alicia C. Marín, David Bernardo, Luis Bujanda, Jesús M. Bañales, Felix Elortza, Javier P. Gisbert, María Chaparro

**Affiliations:** 1 Centro de Investigación Biomédica en Red de Enfermedades Hepáticas y Digestivas (CIBEREHD), Instituto de Investigación Sanitaria Princesa (IIS-Princesa), Hospital Universitario de La Princesa, Universidad Autónoma de Madrid (UAM), 28006 Madrid, Spain; aliciacmarin@gmail.com (A.C.M.); javier.p.gisbert@gmail.com (J.P.G.); mariachs2005@gmail.com (M.C.); 2 Proteomics Platform, CIC bioGUNE, BRTA (Basque Research & Technology Alliance), CIBEREHD, 48160 Derio, Spain; mazkargorta@cicbiogune.es (M.A.); iiloro@cicbiogune.es (I.I.); iescobes@cicbiogune.es (I.E.); felortza@cicbiogune.es (F.E.); 3 Department of Liver and Gastrointestinal Diseases, Biogipuzkoa Health Research Institute—Donostia University Hospital, University of the Basque Country (UPV/EHU), 20014 Donostia-San Sebastian, Spain; ainhoa.lapitzdambolenea@bio-gipuzkoa.eus (A.L.); aarbelaizcossio@gmail.com (A.A.); luis.bujandafernandezdepierola@osakidetza.eus (L.B.); jesusmaria.banalesasurmendi@bio-gipuzkoa.eus (J.M.B.); 4 National Institute for the Study of Liver and Gastrointestinal Diseases (CIBEREHD), Instituto de Salud Carlos III, 28029 Madrid, Spain; 5 Área de Farmacología y Nutrición y Bromatología, Grupo de Investigación de Alto Rendimiento en Fisiopatología del Sistema Digestivo URJC: NeuGut-URJC, Departamento Ciencias Básicas de la Salud, Universidad Rey Juan Carlos, 28922 Madrid, Spain; lorena.ortega@urjc.es; 6 Departamento de Nutrición y Ciencia de los Alimentos, Facultad de Farnmacia, Universidad Complutense de Madrid, 28040 Madrid, Spain; sfernandeztome@ucm.es; 7 Mucosal Immunology Lab, Unit of Excelence Institute of Biomedicine and Molecular Genetics (IBGM), Centro de Investigaciones Biomédicas en Red de Enfermedades Infecciosas (CIBERINFEC), University of Valladolid (CSIC), 47005 Valladolid, Spain; d.bernardo.ordiz@gmail.com; 8 IKERBASQUE, Basque Foundation for Science, 48011 Bilbao, Spain; 9 Department of Biochemistry and Genetics, School of Sciences, University of Navarra, 31009 Pamplona, Spain

**Keywords:** Crohn’s disease, ulcerative colitis, extracellular vesicles, proteomics

## Abstract

The proteomic analysis of serum extracellular vesicles (EVs) could be a useful tool for studying the pathophysiology of Crohn’s disease (CD) and ulcerative colitis (UC), as well as for biomarker discovery. To characterize the proteomic composition of serum EVs in patients with CD and UC to identify biomarkers and molecular pathways associated with pathogenesis and activity. Methods: Serum EVs were enriched and analyzed in patients with quiescent CD, active CD (aCD), quiescent UC, active UC (aUC), and healthy controls (HCs) (n = 30 per group). All groups were matched for age and sex. Disease activity was assessed by ileocolonoscopy and categorized based on the SES-CD (CD) and the endoscopic sub-score of the Mayo Score (UC). EVs were enriched by ul-tracentrifugation, and their size and concentration were determined by nanoparticle tracking analysis. The expression of CD63, CD81, and CD9 was determined using West-ern blotting. Proteomic analysis was performed by label-free nano-LC MS/MS. A total of 324 proteins were identified; 60 showed differential abundance in CD-HC, 34 in UC-HC, and 21 in CD-UC. Regarding disease activity, the abundance of 58 and 32 proteins was altered in aCD-HC and aUC-HC, respectively. Functional analyses revealed that proteins associated with aCD were involved in immune regulation, whereas those linked to aUC were enriched in oxidative stress. We have identified expressed proteins between EVs from patients with CD and UC, depending on the presence of disease, the disease type, and the disease activity. These proteins are potential candidates as disease biomarkers and open new research avenues to better understand these conditions.

## 1. Introduction

Inflammatory bowel diseases (IBDs)—including Crohn’s disease (CD) and ulcerative colitis (UC)—are chronic inflammatory diseases of the gastrointestinal tract with a high burden for both patients and society [1]. There is no curative treatment for IBDs, primarily due to their complex and largely unknown etiology, involving interactions between environmental factors, genetics, diet, the intestinal microbiota, and the immune system [2].

Current assessment methods, primarily based on endoscopy, are considered to be the gold standard for evaluating mucosal activity; however, they are invasive and costly, thus limiting their routine application [3]. This has prompted an increasing interest in non-invasive biomarkers, which may facilitate patient monitoring and improve quality of life [4,5,6].

Extracellular vesicles (EVs) are membrane-bound particles secreted by cells, which play a crucial role in intercellular communication and can encapsulate various biomolecules, including proteins that contribute to disease pathogenesis [7]. These diverse contents endow EVs with biomarker potential for disease diagnosis, prognosis, and monitoring [8]. Despite the gastrointestinal tract being a primary source of human EVs, their specific roles in IBD pathogenesis and correlation with disease types remain poorly characterized. Preliminary studies suggest that EVs modulate immune responses through proteins involved in macrophage activation [9,10].

EVs containing Annexin A1 activate wound repair pathways in mice, and their levels are elevated in the sera of active IBD patients compared to healthy controls (HCs) [11]. This suggests that they could be useful in disease monitoring and as potential therapeutic targets. Additionally, EVs derived from inflamed intestinal sites in IBD patients exhibit distinct protein profiles; EVs from patients with severe inflammation contain increased levels of inflammatory cytokines, while those from patients in endoscopic remission have cytokine levels akin to those from HCs [12]. Notably, these active IBD-derived EVs exert pro-inflammatory effects on colonic epithelial cells in vitro.

The present study analyzed the proteome composition of serum EVs from IBD (CD and UC) patients, with and without intestinal inflammation, and HCs. The aim was to perform a comprehensive proteomic characterization of EVs to discover potential new biomarkers and identify molecular pathways involved in IBD pathogenesis. The focus on serum EVs aimed to overcome the limitations of traditional serum proteomic analyses, which often hinder the identification of low-abundance proteins that could play crucial roles in IBD pathogenesis.

## 2. Results

### 2.1. Characterization of Serum EVs from IBD Patients and HCs

**Figure 1:**
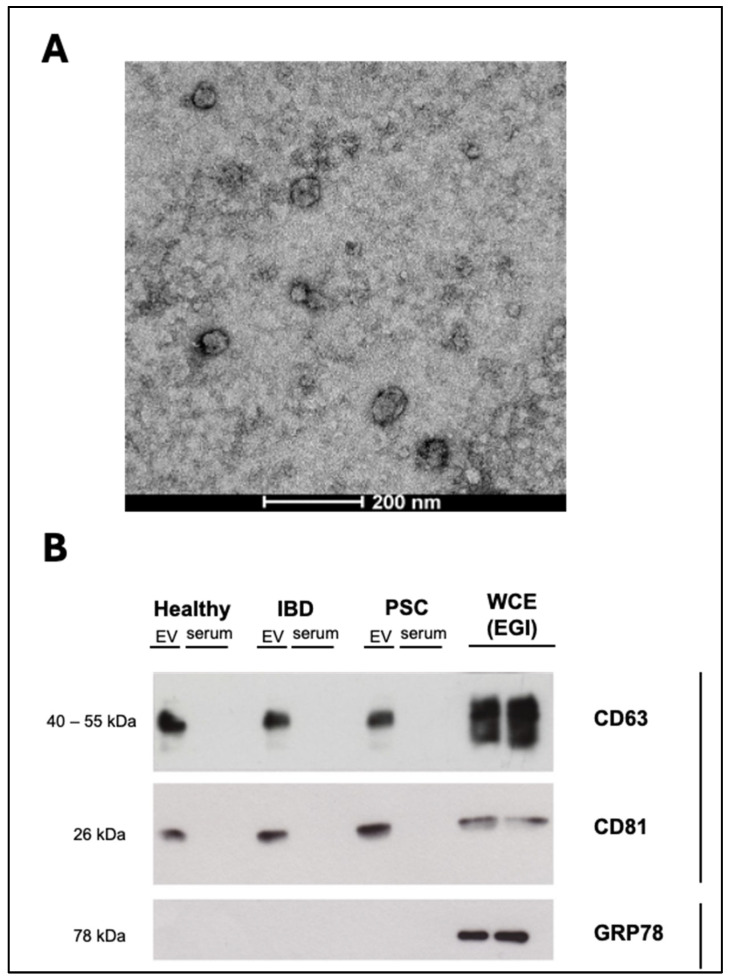
Characterization of serum extracellular vesicles (EVs): (**A**) Transmission electron microscopy image of EVs derived from serum. Scale bar represents 200 nm. (**B**) Western blot analysis using the specific EV markers CD63 and CD81 (positive controls) and GRP78 (negative control) from EVs and serum of healthy and inflammatory bowel disease patients, compared to whole-cell extract of cholangiocytes. IBD: inflammatory bowel disease, PSC: primary sclerosing cholangitis, EV: extracellular vesicle, WCE: whole-cell extract.

**Figure 2:**
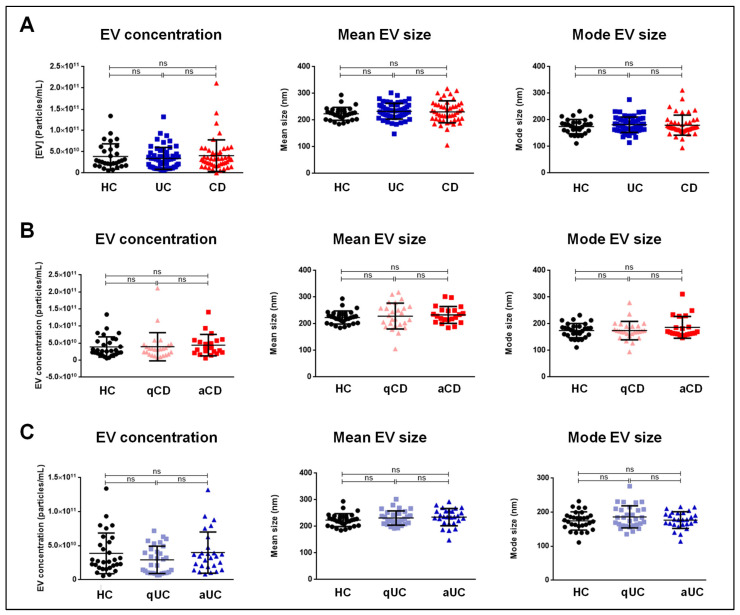
Concentration (particles/mL) and mean and mode size (nm) of serum extracellular vesicles (EVs) in the different study groups: (**A**) Comparison between Crohn’s disease (CD), ulcerative colitis (UC) and healthy controls (HCs) subjects. (**B**) Comparison between active CD (aCD), quiescent CD (qCD) and HCs. (**C**) Comparison between active UC (aUC), quiescent UC (qUC) and HCs. Extracellular vesicle concentration and size were assessed using nanoparticle tracking analysis. ns: not significant.

Serum EVs were characterized by NTA. The vesicles showed round morphology by TEM and expression of the typical EV protein markers CD81, CD63, and CD9, as analyzed by Western blotting (Figure 1 and Figure S1). The size and concentration of EVs from CD or UC patients (active and quiescent) and HCs are shown in Table 1. No significant differences were observed in EV concentration or size across groups (CD vs. UC vs. HCs) (Figure 2A), between active CD (aCD), quiescent CD (qCD), and HCs (Figure 2B), or between active UC (aUC), quiescent UC (qUC), and HCs (Figure 2C).

### 2.2. Proteomic Characterization of Serum EVs from Active and Quiescent UC and CD Patients, and HC

**Figure 3:**
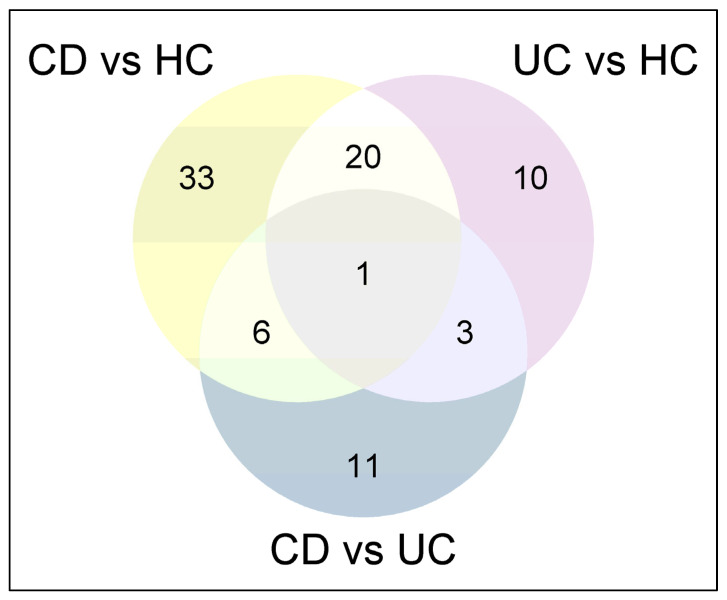
Venn diagram representing the number of overlapping differential abundance serum extracellular vesicle proteins among different comparisons between patients with CD, UC, and HCs. HC: healthy control; CD: Crohn’s disease; UC: ulcerative colitis.

A total of 324 proteins were identified by at least two unique peptides with an FDR < 1% detected in serum EVs (fold-change ratio > 1.5, *p*-value < 0.05). Of these, 60 proteins showed differential abundance between CD and HCs, 34 between UC and HCs, and 21 between CD and UC (Supplementary Table S1). A Venn diagram revealed that 33 proteins were exclusively found in the comparison between CD patients and HCs, 10 proteins between UC patients and HCs, and 11 proteins between CD and UC (Figure 3).

**Figure 4:**
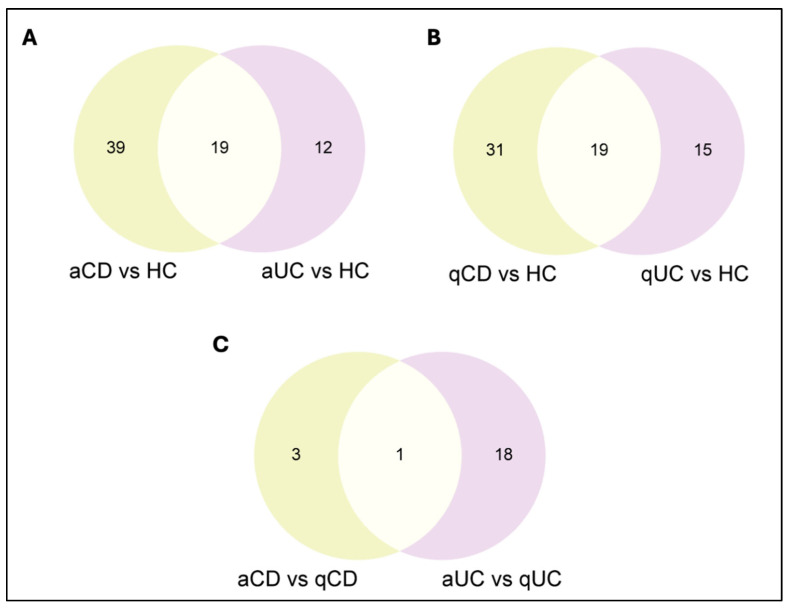
Venn diagram representing the number of overlapping differential abundance serum extracellular vesicle proteins among different study comparisons between different patient groups and healthy controls: (**A**) aCD vs. HCs and aUC vs. HCs. (**B**) qCD vs. HCs and qUC vs. HCs. (**C**) aCD vs. qCD and aUC vs. qUC. HC: healthy control; CD: Crohn’s disease; UC: ulcerative colitis; aCD: active Crohn’s disease; qCD: quiescent Crohn’s disease; aUC: active ulcerative colitis; qUC: quiescent ulcerative colitis.

Regarding disease activity, the abundance of 58 and 31 proteins was altered in aCD and aUC patients compared to HCs, respectively (Figure 4A, Table S2). In contrast, the abundance of 50 proteins was found to be altered in qCD patients and 34 in qUC patients compared to HCs (Figure 4B, Table S3). Notably, only 4 proteins showed differential abundance between aCD and qCD patients, while the abundance of 19 proteins was altered between aUC and qUC (Figure 4C, Table S4). Therefore, these results confirm that the protein content from the EVs is related to disease activity and, indeed, is more pronounced in UC than in CD.

### 2.3. Novel Serum EV Biomarkers for UC and CD, and Their Association with Disease Activity

In UC patients, a total of 14 proteins were identified with potential diagnostic value (*p*-value < 0.05 and ACC ≥ 0.65) either individually or in combination with other proteins. Among them, the abundance of 10 proteins was altered between UC and HCs, and 4 showed classification potential only in combination with other proteins. A summary of these biomarker candidates is presented in Table 2.

In aUC patients, 26 classifiers were identified as potential biomarkers. Of these, 21 were single-protein features, while 5 were multi-protein features. Interestingly, 11 proteins did not show differential expression between aUC and HCs but still exhibited strong classification potential. Table 3 lists only the proteins that were found to have differential abundance in the proteomic analysis.

Regarding the comparison between qUC patients and HCs, the abundance of 42 proteins was found to be altered alone and/or in combination with another molecule. Of these, the abundance of five proteins was altered between the qUC and HC cohorts. Nine molecules presented classification power only in combination with another molecule, thirty-three molecules presented classification power on their own, and seven molecules presented classification power both individually and in combination with another molecule. The proteins that demonstrated good discriminatory power and were also found to be differential in the discovery phase are shown in Table 4.

In relation to CD, 50 out of the 324 evaluated proteins showed differential abundance compared to HCs. Of these, 22 proteins with potential classification power were detected. Out of those 22 proteins, 16 were found to have differential abundance between the CD and HC cohorts. Four molecules possessed classification power only in combination with another molecule, eighteen molecules presented classification power on their own, and five molecules evidenced classification power both individually and in combination with another molecule. Table 5 shows the results only for those proteins that were found to be differential in the proteomic analysis.

The comparison between serum EV samples from aCD patients and those from HCs provided a total of 37 proteins with potential classification power. Out of those 37 proteins, 18 were found to have differential abundance between the aCD and HC cohorts. Three proteins possessed classification power only in combination with another molecule, thirty-four molecules presented classification power on their own, and six molecules presented classification power both individually and in combination with another molecule. Table 6 shows only the proteins that were found to be differential in the proteomic analysis.

In the comparison between qCD patients and HCs, 32 proteins were detected with potential classification power individually and/or in combination with another molecule. Out of those 32 proteins, the abundance of 15 was altered between the aCD and HC cohorts. Four of them had classification power only in combination with another molecule, twenty-eight molecules presented classification power on their own, and twelve molecules presented classification power both individually and in combination with another molecule. Those proteins that demonstrated good discriminatory power and were also found to be differential in the discovery phase are shown in Table 7.

Based on these findings, two subsets of biomarker candidates were selected as candidates for further validation in an independent cohort in the future. The first subset comprised the proteins that exhibited the best accuracy in distinguishing between the different conditions, representing the most robust discriminatory markers (Table 8). The second subset included proteins with good accuracy, albeit with slightly lower ACC values compared to the first group, thus indicating a potential relevance with less discriminative power (Table 9).

### 2.4. Functional Enrichment Analysis of Differential-Abundance Serum EV Proteins Associated with the Disease Activity

To gain a deeper understanding of the proteins potentially associated with disease activity in IBD, and to comprehensively explore the biological significance of differential-abundance proteins in serum EVs from patients with CD and UC in both remission and active phases of the disease, a GO functional enrichment analysis was performed. The GO enrichment analysis bubble plot and the GO enrichment analysis bar plot displayed the top three notably enriched GO terms.

**Figure 5:**
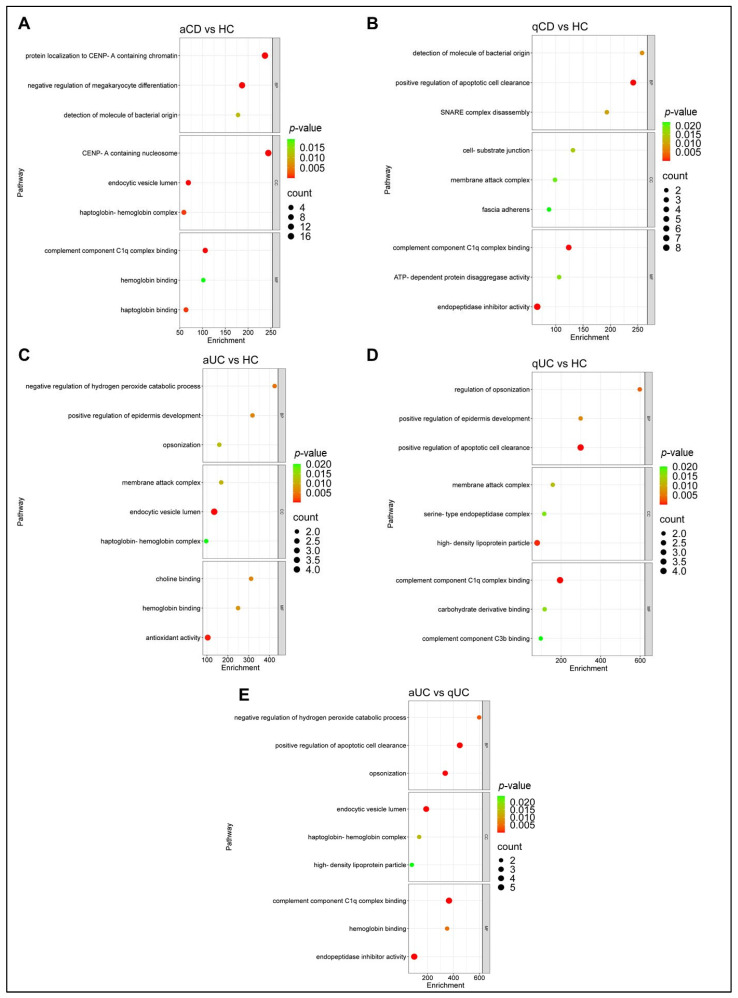
Functional enrichment analysis of differential-abundance serum extracellular vesicle proteins associated with disease activity. Bubble plots represent enriched Gene Ontology (GO) terms for biological processes (BPs), cellular components (CCs), and molecular functions (MFs) in different comparisons: (**A**) aCD vs. HC, (**B**) qCD vs. HC, (**C**) aUC vs. HC, (**D**) qUC vs. HC, (**E**) and aUC vs. qUC. The color intensity of the nodes represents the adjusted *p*-value, and the node size indicates the number of genes. HC: healthy control; CD: Crohn’s disease; UC: ulcerative colitis; aCD: active Crohn’s disease; qCD: quiescent Crohn’s disease; aUC: active ulcerative colitis; qUC: quiescent ulcerative colitis.

The results showed that most of the proteins with great differential expression in serum EVs between aCD patients and HCs were involved in the following biological processes: protein localization to CENP-A-containing chromatin, megakaryocyte differentiation, and detection of molecules of bacterial origin. For the molecular function category, differential proteins were mainly enriched in the complement component C1q complex, hemoglobin binding, and haptoglobin binding; and for the cellular component category, these proteins were enriched in the CENP-A-containing nucleosome, endocytic vesicle lumen, and haptoglobin–hemoglobin complex (Figure 5A). The biological processes category of the GO analysis of qCD patients compared to HCs showed that differential-abundance proteins were significantly enriched in the terms positive regulation of apoptotic cell clearance, detection of molecules of bacterial origin, and SNARE complex disassembly (Figure 5B). For GO cellular component analysis, the top three significantly enriched terms were cell–substrate junction, membrane attack complex, and fascia adherens, and the significantly enriched molecular function terms included complement component C1q complex, ATP-dependent protein disaggregase activity, and endopeptidase inhibitor activity.

Furthermore, GO functional enrichment analysis was conducted on the serum EV proteins with differential abundance between aUC and qUC patients. The main biological processes found in this analysis included negative regulation of hydrogen peroxide catabolic process, regulation of epidermis development, and opsonization (Figure 5C). The main cellular component terms included membrane attack complex, endocytic vesicle lumen, and haptoglobin–hemoglobin complex, while the main molecular function terms included choline binding, hemoglobin binding, and antioxidant activity. The comparison between qCD patients and HCs revealed biological processes related to the regulation of opsonization, regulation of epidermis development, and positive regulation of apoptotic cell clearance (Figure 5D). For the cellular component category, the differential-abundance proteins were mainly enriched in membrane attack complex, serine-type endopeptidase complex, and high-density lipoprotein particle. The main molecular function terms included the complement component C1q complex, carbohydrate derivative binding, and complement component C3b binding.

Finally, in the comparison of UC patients (active vs. quiescent), the GO functional enrichment analysis showed that negative regulation of hydrogen peroxide’s catabolic process, regulation of apoptotic clearance, and opsonization were the main enriched biological processes (Figure 5E). For the cellular component category, the differential-abundance proteins were mainly enriched in the terms endocytic vesicle lumen, haptoglobin–hemoglobin complex, and high-density lipoprotein particle, while in the molecular function category these proteins were enriched in the complement component C1q complex, hemoglobin binding, and endopeptidase inhibitor activity. The functional analysis of the comparison between aCD and qCD could not be performed due to the limited differences between groups (the abundance of only four proteins was altered).

## 3. Discussion

This study provides insights into the profile of circulating EVs in IBD patients, suggesting their potential to reflect disease condition and distinguish between active and quiescent sates. Hence, these results have a great impact, not just in unravelling the basis underlying IBD’s pathogenesis, but in identifying proteins with potential utility as novel biomarkers to aid in the diagnosis and/or monitoring of IBD in the absence of an invasive colonoscopy.

Despite advancements in the clinical management of IBD patients, there is still a lack of useful non-invasive biomarkers that can reliably facilitate the diagnosis and monitoring of disease progression and treatment response. EVs have emerged as key mediators of intercellular communication, carrying biomolecules that influence inflammation and immune responses in the gastrointestinal tract. Evidence suggests that the protein composition of EVs can reflect the inflammatory state of the intestine [13]. In this context, previous studies have pointed to the potential of EVs as non-invasive biomarkers for IBD. One study identified the proteasome subunit alpha type 7 in salivary EVs as a promising candidate to differentiate IBD patients from HCs [14]. However, that study did not establish a correlation between biomarker expression and disease activity, nor could it effectively compare CD and UC due to its limited sample size. Another study analyzed serum exosomes in IBD patients compared to HCs and in a mouse model of acute colitis [15]. Pregnancy zone protein, known for its immunosuppressive properties, was significantly increased in serum exosomes both in IBD and in mouse colitis. However, like the previous studies, this research did not distinguish between CD and UC patients, and the small sample size limited the findings. Collectively, these studies highlight the premise of EVs as biomarkers for IBD while emphasizing the need for larger, more comprehensive investigations to clarify their clinical utility.

This study is unique as it is the first to isolate serum EVs from IBD patients and HCs specifically distinguishing, among IBD patients, between CD and UC and between patients with and without intestinal inflammation. By analyzing EVs’ protein profiles, we identified potential biomarkers associated with IBD presence and disease activity. Our findings are consistent with those of previous studies that have demonstrated that EVs carry biomolecules reflective of inflammatory processes in the gut. Notably, we observed significant differences in the proteomic composition of EVs between active disease in CD and UC compared to HCs, which points to the potential value of EVs as non-invasive biomarkers for disease activity.

Our findings revealed no significant differences in EV size or concentration across the different groups, including comparisons between the active and quiescent phases of the disease. These data suggest that the total number of EVs does not vary significantly between IBD patients and HCs. Therefore, functional changes in EV composition, rather than their quantity, may better reflect disease activity.

There is a strong association between disease activity and the differential abundance of specific proteins, underscoring distinct molecular alterations in aCD and aUC compared to HCs. These differences suggest that the molecular alterations accompanying disease exacerbations differ between CD and UC. These findings are relevant, as they suggest that targeted therapeutic strategies could be designed based on these specific proteomic signatures, potentially leading to more personalized treatment approaches.

Additionally, we identified two distinct subsets of protein biomarkers capable of distinguishing CD and UC from HCs, which deserve further validation in an independent cohort. The first subset showed the highest discriminative power, emerging as the most reliable set of markers. However, the second subset demonstrated moderate accuracy and exhibited slightly lower discriminative capability. Despite this, these proteins may still hold significant potential for disease characterization.

Functional enrichment analysis of the differential-abundance proteins in serum EVs has provided valuable insights into the biological processes associated with disease activity in IBD patients. Differential serum EV proteins in aCD were enriched in biological processes such as protein localization to CENP-A-containing chromatin and megakaryocyte differentiation. These processes, which are fundamental for genetic maintenance [16] and the formation of cells involved in hemostasis [17], have not been previously associated with IBD. Notably, the molecular functions of these proteins are enriched in components like the complement component C1q complex, which plays a pivotal role in immune regulation, and in binding activities associated with hemoglobin and haptoglobin, indicating systemic effects of inflammation.

In aUC, differential serum EV proteins were involved in processes such as the negative regulation of hydrogen peroxide catabolic processes, epidermis development, and opsonization, suggesting altered oxidative stress responses and immune modulation during active disease [18]. Previous studies have shown that hydrogen peroxide levels are increased in the non-inflamed colonic epithelium of UC patients, evidencing the role of hydrogen peroxide in both the pathogenesis and relapse of this debilitating form of IBD [19,20]. Additionally, genetically engineered mice that are unable to neutralize colonic hydrogen peroxide [glutathione (GSH) peroxidase-knockout mice] develop colitis analogous to human UC [21]. This indicates that hydroxide peroxide generated in colonic epithelial cells can diffuse extracellularly and initiate colonic inflammation. At the molecular level, significant functions such as choline binding, hemoglobin binding, and antioxidant activity suggest potential dysregulation of oxidative pathways that may be directly associated with disease activity in aUC.

Although the findings described herein pointing to candidate biomarkers for IBD are promising, their preliminary nature is the main limitation of our study. Future studies in larger and independent cohorts of patients will be necessary to validate these biomarkers and evaluate their predictive ability for disease activity. Moreover, further functional studies thoroughly elucidating the biological role of the differential-abundance serum EV proteins in the pathogenesis of IBD will be critical for advancing our understanding of these diseases.

This study has several strengths. It is the first study to comprehensively characterize the proteomic profile of serum-derived EVs in CD and UC, in both active and quiescent disease states, and HCs, with the aim of identifying proteins associated with disease activity. Furthermore, the inclusion of a large, well-defined cohort of 150 participants (30 aCD, 30 qCD, 30 aUC, 30 qUC, and 30 HCs) provided a robust dataset for analysis, significantly enhancing this study’s statistical power and serving as a starting point for future validation studies.

## 4. Material and Methods

### 4.1. Study Design

A total of 150 participants were included in the study: 30 quiescent CD patients (qCD), 30 active CD patients (aCD), 30 quiescent UC patients (qUC), 30 active UC patients (aUC), and 30 HCs. The demographic and clinical characteristics of the study population are shown in Table 10. Serum samples from subjects who met the inclusion criteria were provided by the Gastrointestinal Biologic Samples Collection of Dr. Javier P. Gisbert (Reg. C.0003482). The Ethics Committee of Hospital Universitario de La Princesa approved the study protocol.

### 4.2. Subject Recruitment

IBD patients (CD or UC) with active or inactive endoscopic disease, undergoing a colonoscopy indicated by medical criteria, were considered. The CD patients had luminal disease (CD patients with perianal disease were not included in the study). Only patients with complete ileocolonoscopy were considered. Patients were classified into active or quiescent IBD according to endoscopic findings. HCs included subjects undergoing colonoscopy for colorectal cancer surveillance, changes in bowel habit, or rectal bleeding. Only individuals with a macroscopically and histologically normal intestine and no evidence of disease were selected. The exclusion criteria for both groups included pregnancy, active infection, neoplasia, or any chronic condition that could affect the results.

### 4.3. Data Collection

The variables included in the database were IBD type (location and behavior), age at IBD diagnosis, time of disease evolution, smoking habit, surgical interventions due to IBD, IBD treatment, and clinical and endoscopic disease activity.

### 4.4. Endoscopic IBD Activity

Experienced gastroenterologists performed the ileocolonoscopies and graded the findings according to the Simple Endoscopic Score index for CD (SES-CD) and the Mayo endoscopic sub-score for UC. An SES-CD score between 0 and 2 was considered to represent inactive CD. Regarding UC activity, Mayo endoscopic sub-scores of 0 and 1 were considered to represent inactive UC.

### 4.5. Serum EV Isolation

Serum samples (1 mL aliquots) were thawed at room temperature and processed using a series of centrifugations at 4 °C. First, serum was centrifuged to remove small cell debris, followed by ultracentrifugation at 100,000 × *g* for 75 min to pellet the EVs. The EV pellet was washed and subjected to a second ultracentrifugation under the same conditions. The final EV pellet was resuspended in 20 µL of phosphate-buffered saline (PBS) for immediate use or stored at −80 °C for further experiments.

### 4.6. Transmission Electron Microscopy (TEM)

For the characterization of EVs, PBS-resuspended EV isolates were negatively stained and evaluated by TEM. EV samples were directly adsorbed onto a glow-discharged (60 seg low discharging using a PELCO easy-glow device) carbon-coated copper grid (300 mesh). Afterwards, the grids were fixed with 2% paraformaldehyde (PFA) in 0.2 M PBS (pH 7.4) for 20 min and washed with distilled water. Then, contrast staining was performed by incubating the grids with 4% uranyl acetate at 4 °C for 15 min. TEM images were obtained by using a TECNAI G2 20 C-TWIN high-resolution transmission electron microscope (FEI, Donostia-San Sebastian, Spain), at an acceleration voltage of 200 kV.

### 4.7. Distribution and Concentration of EVs

The size distribution and concentration of the EVs were measured by nanoparticle tracking analysis (NTA) using a NanoSight LM10 system (Malvern, UK). Post-acquisition settings for NTA were standardized for all samples. Each video was analyzed to determine the mode vesicle size and EV concentration.

### 4.8. Western Blot Analysis

Protein expression in the EV samples was analyzed by immunoblotting. Positive (CD63 and CD8) and negative (GRP78) EV markers were evaluated.

### 4.9. Mass Spectrometry Analysis and Protein Identification

The samples were processed with an extraction buffer (7 M urea, 2 M thiourea, 4% CHAPS, and 100 mM DTT) and incubated for 30 min with agitation. Next, Filter-Aided Sample Preparation (FASP) was performed for the digestion of proteins. After digestion, peptides were recovered from the filter units and subjected to ethyl acetate. After careful removal of the last upper ethyl acetate layer, the samples were speed-vacuumed in an RVC2 25 speedvac concentrator (Christ). The samples were further desalted using stage-tip C18 microcolumns (Zip-tip, Millipore, Burlington, MA, USA) and resuspended in 0.1% FA prior to mass spectrometry analysis.

Peptide separation was conducted in a nanoACQUITY UPLC system (Waters, Milford, MA, USA) coupled with an LTQ Orbitrap XL (Thermo Electron, Waltham, MA, USA) and/or Synapt G2 Si (Waters) mass spectrometer. A linear gradient of 3–50% acetonitrile over 120 min was used for peptide elution. Peptides were identified using Mascot v2.1 (Matrix Science) through Proteome Discoverer 1.4 (Thermo Electron), with carbamidomethylation of cysteines as a fixed modification and oxidation of methionines as a variable modification. Searches were conducted against the Uniprot/Swissprot database, with a decoy search to estimate the false discovery rate (FDR). Only peptides with an FDR < 1% were selected.

For label-free differential protein expression analysis, Progenesis LC-MS (Nonlinear Dynamics) was used. Raw files were imported from both Orbitrap and Synapt runs, with one run designated as the reference for aligning precursor masses in all other samples. The raw abundances of each feature were automatically normalized against the reference run and logarithmized. Samples were grouped according to the comparisons being performed. A peak list containing the information of these significantly different features was generated and exported to the Mascot search engine (Matrix Science Ltd., Boston, MA, USA) for the identification of peptides. The list of identified peptides was imported in Progenesis LC-MS, and the previously quantified features were matched to the corresponding peptides. Only proteins with at least two quantified non-conflicting peptides were selected. Proteins with a *p*-value < 0.05 and ratio > 1.5 were considered to be significantly deregulated.

### 4.10. Functional Analysis

Gene Ontology (GO) enrichment analysis was carried out using the Database for Annotation, Visualization, and Integrated Discovery (DAVID) online tool (http://david.abcc.ncifcrf.gov/summary.jsp, accessed on 25 December 2024). DAVID is a GO term annotation and enrichment analysis tool used to highlight the most relevant GO terms associated with a given gene list. Fisher’s exact test was used to determine whether the proportion of genes considered to belong to certain GO terms or categories differed significantly between the dataset and the background. Biological process, molecular function, and cellular component categories were assessed, and only GO terms enriched with an FDR < 5% were considered for comparison and discussion.

### 4.11. Statistical Analysis

Quantitative variables are presented as means and standard deviations. Categorical variables are presented as numbers of events and percentages. The Kolmogorov–Smirnov test was used to determine the normality of data distribution for quantitative variables. Differences in this type of variable between groups were analyzed by Student’s *t* test or the Wilcoxon rank-sum test according to whether the variables were normally distributed or not. For categorical variables, differences between groups were assessed using the chi-squared test. Differences were considered significant when *p* < 0.05.

For biomarker discovery, the dataset with all of the variables from the individual patients was analyzed by Anaxomics Biotech (http://www.anaxomics.com, accessed on 25 December 2024) using a data mining approach, employing the algorithm Balanced_Accuracy [22]. Cross-validated balanced accuracy (ACC) was used as a classifier optimization measure together with the cross-validated *p*-value. In order to prioritize the generalization capability of the conclusion, a K-fold validation analysis was performed, yielding cross-validated quality measures (such as accuracy) for each biomarker [23].

Functional and pathway enrichment analysis was conducted using DAVID to perform GO enrichment analysis using SRplot [24].

## 5. Conclusions

In conclusion, this research highlights the potential role of EVs in IBD’s pathogenesis. The proteomic characterization of serum EVs offers valuable insights into the underlying pathogenic mechanisms and provides information to identify specific biomarkers of disease activity. These findings represent a starting point for studying the role of EVs in IBD; after validation of these findings in future studies, the incorporation of these biomarkers into clinical practice could significantly improve the monitoring and management of IBD, paving the way for more personalized treatment strategies.

## Figures and Tables

**Table 1:** Concentration and size of serum extracellular vesicles in each study group. HC: healthy control; CD: Crohn’s disease; UC: ulcerative colitis; aCD: active Crohn’s disease; qCD: quiescent Crohn’s disease; aUC: active ulcerative colitis; qUC: quiescent ulcerative colitis; SD: standard deviation.

Patient	Concentration (Particles/mL) Mean ± SD	Size	
		Mean (nm) Mean ± SD	Mode (nm) Mean ± SD
HC	3.88 × 10^10^ ± 2.99 × 10^10^	224 ± 24	174 ± 27
UC	3.43 × 10^10^ ± 2.57 × 10^10^	233 ± 29	182 ± 29
CD	4.17 × 10^10^ ± 3.70 × 10^10^	231 ± 41	180 ± 38
aCD	4.42 × 10^10^ ± 3.02 × 10^10^	235 ± 33	177 ± 25
qCD	3.96 × 10^10^ ± 2.01 × 10^10^	232 ± 27	186 ± 33
aUC	3.99 × 10^10^ ± 3.14 × 10^10^	233 ± 31	187 ± 41
qUC	2.93 × 10^10^ ± 4.15 × 10^10^	229 ± 48	174 ± 35

**Table 2:** Summary of proteins with potential as biomarkers to differentiate between ulcerative colitis and healthy controls.

Feature	Protein Name	Balanced Cross-Validated Accuracy
ECM1_HUMAN	Extracellular matrix protein 1	0.74
CO3_HUMAN	Complement C3	0.72
F13A_HUMAN	Coagulation factor XIII A chain	0.72
K1C10_HUMAN	Keratin, type I cytoskeletal 10	0.71
CO9_HUMAN	Complement component C9	0.70
THRB_HUMAN	Prothrombin	0.67
LRP1B_HUMAN	Low-density lipoprotein receptor-related protein 1B	0.67
SAA2_HUMAN,ITIH1_HUMAN	Serum amyloid A-2 protein; inter-alpha-trypsin inhibitor heavy chain H1	0.79
CO3_HUMAN,CO6_HUMAN	Complement C3; complement component C6	0.72
F13A_HUMAN,CO6_HUMAN	Coagulation factor XIII A chain; complement component C6	0.72
SAA2_HUMAN,LRP1B_HUMAN	Serum amyloid A-2 protein; low-density lipoprotein receptor-related protein 1B	0.72
SAA2_HUMAN,CO6_HUMAN	Serum amyloid A-2 protein; complement component C6	0.72

**Table 3:** Summary of proteins with potential as biomarkers to differentiate between active ulcerative colitis and healthy controls.

Feature	Protein Name	Balanced Cross-Validated Accuracy
SAA2_HUMAN	Serum amyloid A-2 protein	0.83
F13A_HUMAN	Coagulation factor XIII A chain	0.81
ECM1_HUMAN	Extracellular matrix protein 1	0.79
ALBU_HUMAN	Serum albumin	0.75
LBP_HUMAN	Lipopolysaccharide-binding protein	0.72
CO6_HUMAN	Complement component C6	0.72
CO9_HUMAN	Complement component C9	0.72
CO3_HUMAN	Complement C3	0.69
TRFE_HUMAN	Serotransferrin	0.68
TPP2_HUMAN	Tripeptidyl-peptidase 2	0.65
F13A_HUMAN,AACT_HUMAN	Coagulation factor XIII A chain; alpha-1-antichymotrypsin	0.88
F13A_HUMAN,SAA2_HUMAN	Coagulation factor XIII A chain; serum amyloid A-2 protein	0.84
F13A_HUMAN,CO9_HUMAN	Coagulation factor XIII A chain; complement component C9	0.83

**Table 4:** Summary of proteins with potential as biomarkers to differentiate between quiescent ulcerative colitis and healthy controls.

Feature	Protein Name	Balanced Cross-Validated Accuracy
C4BPB_HUMAN	C4b-binding protein beta chain	0.74
SAA2_HUMAN	Serum amyloid A-2 protein	0.72
PROS_HUMAN	Vitamin K-dependent protein S	0.65
K1C10_HUMAN,C4BPB_HUMAN	Keratin, type I cytoskeletal 10; C4b-binding protein beta chain	0.80
K1C10_HUMAN,CO4A_HUMAN	Keratin, type I cytoskeletal 10; complement C4-A	0.75

**Table 5:** Summary of proteins with potential as biomarkers to differentiate between Crohn’s disease and healthy controls.

Feature	Protein Name	Balanced Cross-Validated Accuracy
F13A_HUMAN	Coagulation factor XIII A chain	0.81
THRB_HUMAN	Prothrombin	0.78
CO3_HUMAN	Complement C3	0.77
ITIH2_HUMAN	Inter-alpha-trypsin inhibitor heavy chain H2	0.73
ECM1_HUMAN	Extracellular matrix protein 1	0.72
H2B1B_HUMAN;H2B1A_HUMAN	Histone H2B type 1-B; histone H2B type 1-A	0.72
APOD_HUMAN	Apolipoprotein D	0.69
CFAH_HUMAN	Complement factor H	0.69
MRCKB_HUMAN	Serine/threonine-protein kinase MRCK beta	0.68
CO4B_HUMAN	Complement C4-B	0.67
THIO_HUMAN	Thioredoxin	0.67
H4_HUMAN	Histone H4	0.67
FIBB_HUMAN	Fibrinogen beta chain	0.66
TCO1_HUMAN	Transcobalamin-1	0.66
F13A_HUMAN,THRB_HUMAN	Coagulation factor XIII A chain; prothrombin	0.85
SAA2_HUMAN,THRB_HUMAN	Serum amyloid A-2 protein; prothrombin	0.80
THRB_HUMAN,MRCKB_HUMAN	Prothrombin; serine/threonine-protein kinase MRCK beta	0.79
SAA2_HUMAN,MRCKB_HUMAN	Serum amyloid A-2 protein; serine/threonine-protein kinase MRCK beta	0.76

**Table 6:** Summary of proteins with potential as biomarkers to differentiate between active Crohn’s disease and healthy controls.

Feature	Protein Name	Balanced Cross-Validated Accuracy
F13A_HUMAN	Coagulation factor XIII A chain	0.87
THRB_HUMAN	Prothrombin	0.82
CO3_HUMAN	Complement C3	0.77
MRCKB_HUMAN	Serine/threonine-protein kinase MRCK beta	0.77
K22E_HUMAN	Keratin, type II cytoskeletal 2 epidermal	0.75
APOD_HUMAN	Apolipoprotein D	0.73
BPIB1_HUMAN	BPI fold-containing family B member 1	0.73
ECM1_HUMAN	Extracellular matrix protein 1	0.73
ALBU_HUMAN	Serum albumin	0.72
LRP1_HUMAN	Prolow-density lipoprotein receptor-related protein 1	0.72
C1R_HUMAN	Complement C1r subcomponent	0.70
H2B1B_HUMAN;H2B1A_HUMAN	Histone H2B type 1-B; histone H2B type 1-A	0.70
LBP_HUMAN	Lipopolysaccharide-binding protein	0.70
SAA2_HUMAN	Serum amyloid A-2 protein	0.70
MASP1_HUMAN	Mannan-binding lectin serine protease 1	0.68
IGHG4_HUMAN	Immunoglobulin heavy constant gamma 4	0.67
PROP_HUMAN	Properdin	0.67
F13A_HUMAN,THRB_HUMAN	Coagulation factor XIII A chain; prothrombin	0.93
F13A_HUMAN,SAA2_HUMAN	Coagulation factor XIII A chain; serum amyloid A-2 protein	0.87
SAA2_HUMAN,S10A9_HUMAN	Serum amyloid A-2 protein; protein S100-A9	0.75

**Table 7:** Summary of proteins with potential as biomarkers to differentiate between quiescent Crohn’s disease and healthy controls.

Feature	Protein Name	Balanced Cross-Validated Accuracy
F13A_HUMAN	Coagulation factor XIII A chain	0.80
ECM1_HUMAN	Extracellular matrix protein 1	0.79
THRB_HUMAN	Prothrombin	0.78
CO4A_HUMAN	Complement C4-A	0.74
C4BPB_HUMAN	C4b-binding protein beta chain	0.73
TCO1_HUMAN	Transcobalamin-1	0.73
ARP3_HUMAN	Actin-related protein 3	0.71
CO4B_HUMAN	Complement C4-B	0.71
SAA2_HUMAN	Serum amyloid A-2 protein	0.71
MRCKB_HUMAN	Serine/threonine-protein kinase MRCK beta	0.70
CO3_HUMAN	Complement C3	0.68
CFAH_HUMAN	Complement factor H	0.66
APOC3_HUMAN	Apolipoprotein C-III	0.66
SAA2_HUMAN,THRB_HUMAN	Serum amyloid A-2 protein; prothrombin	0.81
CFAI_HUMAN,FIBB_HUMAN	Complement factor I; fibrinogen beta chain	0.78

**Table 8:** Subset of proteins with the best accuracy to differentiate between the different study groups. The blue color scale represents values of balanced cross-validated accuracy.

			Balanced Cross-Validated Accuracy UC			Balanced Cross-Validated Accuracy CD		
Feature	Components	Peptide Count	UC vs. HC	aUC vs. HC	qUC vs. HC	CD vs. HC	aCD vs. HC	qCD vs. HC
F13A_HUMAN	F13A_HUMAN	8	0.72	0.81		0.81	0.87	0.80
SAA2_HUMAN	SAA2_HUMAN	2		0.83	0.72		0.70	0.71
C4BPB_HUMAN	C4BPB_HUMAN	12			0.74			0.73
CO9_HUMAN	CO9_HUMAN	17	0.70	0.72				
THRB_HUMAN	THRB_HUMAN	24				0.78	0.82	0.78
F13A_HUMAN,THRB_HUMAN	F13A_HUMAN	8				0.85	0.93	
	THRB_HUMAN	24						
THRB_HUMAN,K1C14_HUMAN	THRB_HUMAN	24				0.81		0.88
	K1C14_HUMAN	21						
SAA2_HUMAN,S10A9_HUMAN	SAA2_HUMAN	2					0.75	
	S10A9_HUMAN	3						
SAA2_HUMAN,THRB_HUMAN	SAA2_HUMAN	2			0.79	0.80		0.81
	THRB_HUMAN	24						
F13A_HUMAN,AACT_HUMAN	F13A_HUMAN	8		0.88				
	AACT_HUMAN	4						
F13A_HUMAN,SAA2_HUMAN	F13A_HUMAN	8		0.84			0.87	
	SAA2_HUMAN	2						
F13A_HUMAN,CO9_HUMAN	F13A_HUMAN	8		0.83				
	CO9_HUMAN	17						
K1C10_HUMAN,C4BPB_HUMAN	K1C10_HUMAN	26			0.80			
	C4BPB_HUMAN	12						

HC: healthy control; CD: Crohn’s disease; UC: ulcerative colitis; aCD: active Crohn’s disease; qCD: quiescent Crohn’s disease; aUC: active ulcerative colitis; qUC: quiescent ulcerative colitis.

**Table 9:** Subset of proteins with good accuracy to differentiate between the different study groups. The blue color scale represents the balanced cross-validated accuracy values.

			Balanced Cross-Validated Accuracy UC			Balanced Cross-Validated Accuracy CD		
Feature	Components	Peptide Count	UC vs. HC	aUC vs. HC	qUC vs. HC	CD vs. HC	aCD vs. HC	qCD vs. HC
ECM1_HUMAN	ECM1_HUMAN	11	0.74	0.79		0.72	0.73	0.79
CO6_HUMAN	CO6_HUMAN	17		0.72				
CO3_HUMAN	CO3_HUMAN	103				0.77	0.77	
K22E_HUMAN	K22E_HUMAN	18					0.75	
BPIB1_HUMAN	BPIB1_HUMAN	13					0.73	
CO4A_HUMAN	CO4A_HUMAN	86						0.74
TCO1_HUMAN	TCO1_HUMAN	3						0.73
K1C15_HUMAN	K1C15_HUMAN	14						0.71
ARP3_HUMAN	ARP3_HUMAN	3						0.71
SAA2_HUMAN,CO6_HUMAN	SAA2_HUMAN	2	0.72					
	CO6_HUMAN	17						
F13A_HUMAN,CO6_HUMAN	CO6_HUMAN	17	0.72					
	F13A_HUMAN	8						
K1C10_HUMAN,CO4A_HUMAN	K1C10_HUMAN	26			0.75			
	CO4A_HUMAN	86						

HC: healthy control; CD: Crohn’s disease; UC: ulcerative colitis; aCD: active Crohn’s disease; qCD: quiescent Crohn’s disease; aUC: active ulcerative colitis; qUC: quiescent ulcerative colitis.

**Table 10:** Demographic and clinical characteristics of the study population.

Variables	aCD	qCD	aUC	qUC	HC
Gender (female), n	12	15	12	18	18
Age, years (mean ± SD)	42 ± 9	47 ± 14	48 ± 15	52 ± 13	51 ± 13
CD severity (SES-CD), n					
Remission		27			
Mild	11				
Moderate	8				
Severe	2				
UC severity (Mayo endoscopic sub-score), n					
Remission				21	
Mild				9	
Moderate			12		
Severe			16		

HC: healthy control; CD: Crohn’s disease; UC: ulcerative colitis; aCD: active Crohn’s disease; qCD: quiescent Crohn’s disease; aUC: active ulcerative colitis; qUC: quiescent ulcerative colitis; SES-CD: Simple Endoscopic Score index for CD.

## Data Availability

All proteomics data generated for this study have been made publicly available through the specialized repository, PRIDE, with the following identifier: PXD057391.

## Supplementary Materials

The following supporting information can be downloaded at: www.mdpi.com/article/10.3390/ijms26020526/s1.

## Abbreviations

ACC	Cross-validated balanced accuracy
aCD	Active Crohn’s disease
aUC	Active ulcerative colitis
CD	Crohn’s disease
DAVID	Database for Annotation, Visualization, and Integrated Discovery
EVs	Extracellular vesicles
FDR	False discovery rate
GO	Gene Ontology
HC	Healthy control
IBD	Inflammatory bowel disease
NTA	Nanoparticle tracking analysis
qCD	Quiescent Crohn’s disease
qUC	Quiescent ulcerative colitis
SES-CD	Simple Endoscopic Score index for Crohn’s disease
UC	Ulcerative colitis
